# High intensity interval training as a novel treatment for impaired awareness of hypoglycaemia in people with type 1 diabetes (HIT4HYPOS): a randomised parallel-group study

**DOI:** 10.1007/s00125-023-06051-x

**Published:** 2023-11-27

**Authors:** Catriona M. Farrell, Alison D. McNeilly, Simona Hapca, Paul A. Fournier, Timothy W. Jones, Andrea Facchinetti, Giacomo Cappon, Daniel J. West, Rory J. McCrimmon

**Affiliations:** 1 Division of Systems Medicine, School of Medicine, University of Dundee, Dundee, UK; 2 Computing Science and Mathematics, Faculty of Natural Sciences, University of Stirling, Stirling, UK; 3 University of Western Australia, Perth, WA Australia; 4 Department of Information Engineering, University of Padova, Padova, Italy; 5 Population Health Sciences Institute, Faculty of Medical Science, Newcastle University, Newcastle upon Tyne, UK

**Keywords:** Behaviour, Counter-regulation, Diabetes, Exercise, Habituation, Hypoglycaemia, Impaired awareness

## Abstract

**Aims/hypothesis:**

Impaired awareness of hypoglycaemia (IAH) in type 1 diabetes may develop through a process referred to as habituation. Consistent with this, a single bout of high intensity interval exercise as a novel stress stimulus improves counterregulatory responses (CRR) to next-day hypoglycaemia, referred to as dishabituation. This longitudinal pilot study investigated whether 4 weeks of high intensity interval training (HIIT) has sustained effects on counterregulatory and symptom responses to hypoglycaemia in adults with type 1 diabetes and IAH.

**Methods:**

HIT4HYPOS was a single-centre, randomised, parallel-group study. Participants were identified using the Scottish Diabetes Research Network (SDRN) and from diabetes outpatient clinics in NHS Tayside, UK. The study took place at the Clinical Research Centre, Ninewells Hospital and Medical School, Dundee, UK. Participants were aged 18–55 years with type 1 diabetes of at least 5 years’ duration and HbA_1c_ levels <75 mmol/mol (<9%). They had IAH confirmed by a Gold score ≥4, modified Clarke score ≥4 or Dose Adjustment For Normal Eating [DAFNE] hypoglycaemia awareness rating of 2 or 3, and/or evidence of recurrent hypoglycaemia on flash glucose monitoring. Participants were randomly allocated using a web-based system to either 4 weeks of real-time continuous glucose monitoring (RT-CGM) or RT-CGM+HIIT. Participants and investigators were not masked to group assignment. The HIIT programme was performed for 20 min on a stationary exercise bike three times a week. Hyperinsulinaemic–hypoglycaemic (2.5 mmol/l) clamp studies with assessment of symptoms, hormones and cognitive function were performed at baseline and after 4 weeks of the study intervention. The predefined primary outcome was the difference in hypoglycaemia-induced adrenaline (epinephrine) responses from baseline following RT-CGM or RT-CGM+HIIT.

**Results:**

Eighteen participants (nine men and nine women) with type 1 diabetes (median [IQR] duration 27 [18.75–32] years) and IAH were included, with nine participants randomised to each group. Data from all study participants were included in the analysis. During the 4 week intervention there were no significant mean (SEM) differences between RT-CGM and RT-CGM+HIIT in exposure to level 1 (28 [7] vs 22 [4] episodes, *p*=0.45) or level 2 (9 [3] vs 4 [1] episodes, *p*=0.29) hypoglycaemia. The CGM-derived mean glucose level, SD of glucose and glucose management indicator (GMI) did not differ between groups. During the hyperinsulinaemic–hypoglycaemic clamp studies, mean (SEM) change from baseline was greater for the noradrenergic responses (RT-CGM vs RT-CGM+HIIT: −988 [447] vs 514 [732] pmol/l, *p*=0.02) but not the adrenergic responses (–298 [687] vs 1130 [747] pmol/l, *p*=0.11) in those participants who had undergone RT-CGM+HIIT. There was a benefit of RT-CGM+HIIT for mean (SEM) change from baseline in the glucagon CRR to hypoglycaemia (RT-CGM vs RT-CGM+HIIT: 1 [4] vs 16 [6] ng/l, *p*=0.01). Consistent with the hormone response, the mean (SEM) symptomatic response to hypoglycaemia (adjusted for baseline) was greater following RT-CGM+HIIT (RT-CGM vs RT-CGM+HIIT: −4 [2] vs 0 [2], *p*<0.05).

**Conclusions/interpretation:**

In this pilot clinical trial in people with type 1 diabetes and IAH, we found continuing benefits of HIIT for overall hormonal and symptomatic CRR to subsequent hypoglycaemia. Our findings also suggest that HIIT may improve the glucagon response to insulin-induced hypoglycaemia.

**Trial registration:**

ISRCTN15373978.

**Funding:**

Sir George Alberti Fellowship from Diabetes UK (CMF) and the Juvenile Diabetes Research Foundation.

**Graphical Abstract:**

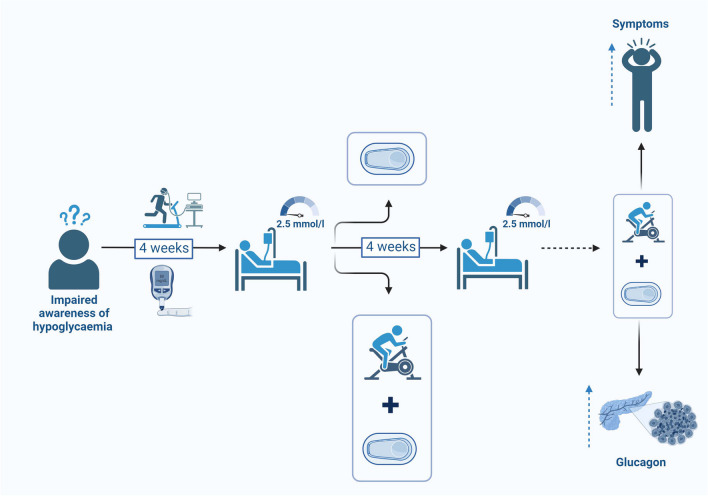

**Supplementary Information:**

The online version contains peer-reviewed but unedited supplementary material available at 10.1007/s00125-023-06051-x.


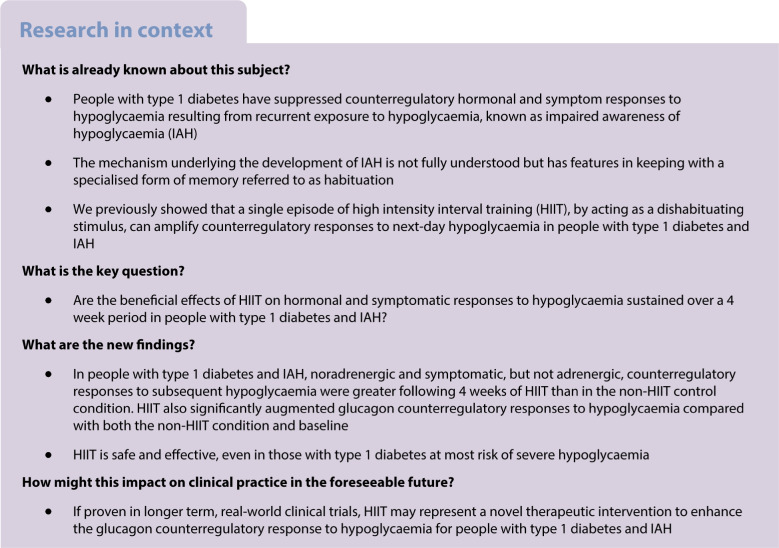


## Introduction

Approximately 25% of people with type 1 diabetes have impaired awareness of hypoglycaemia (IAH), defined as a ‘diminished ability to perceive the onset of acute hypoglycaemia’ [1], which increases their risk of severe hypoglycaemia [2]. IAH is primarily considered to result from repeated exposure to hypoglycaemia. Antecedent hypoglycaemia in people with [3] or without [4] type 1 diabetes leads to suppression of both hormonal and symptomatic responses during hypoglycaemia, while strict avoidance of hypoglycaemia restores counterregulatory responses (CRR) [5]. In this context, strict hypoglycaemia avoidance has remained the primary means by which hypoglycaemia awareness can be improved, historically through relaxation of glucose control and more recently through educational interventions or the use of newer insulins and novel technologies [6]. However, despite this multiplicity of approaches the prevalence of IAH has not significantly improved in the last two to three decades [7].

A number of features of an individual’s response to repeated hypoglycaemia are in keeping with an innate process called habituation. Habituation is a form of adaptive memory, in which there is a reduction in the psychological, behavioural or physiological responses to a stimulus as a result of repeated or prolonged exposure [8, 9]. To test the hypothesis that IAH develops as a habituated response, we recently conducted a proof-of-concept study in people with type 1 diabetes and IAH [10]. In this study we sought to determine whether IAH could be at least temporarily improved by the introduction of a single novel stress stimulus, a phenomenon referred to as dishabituation, a key feature of habituation. Consistent with our hypothesis, we found that a single 20 min bout of high intensity interval exercise significantly augmented the hormonal and symptomatic cognitive CRR to subsequent hypoglycaemia in people with long-duration type 1 diabetes and IAH [10]. However, while a single episode of high intensity interval exercise could at least temporarily improve the counterregulatory defect induced by recurrent hypoglycaemia, it remains possible that individuals may over time also adapt to this stimulus (referred to as habituation to the dishabituating stimulus). Therefore, the aim of this longitudinal pilot study was to investigate whether a 4 week programme of intermittent high intensity interval training (HIIT) would improve counterregulatory hormone and symptom responses and hypoglycaemia awareness in adults with type 1 diabetes and IAH.

## Methods

The full study protocol has been published [11].

### Participant recruitment and randomisation

HIT4HYPOS was a single-centre, randomised, parallel-group study carried out at Ninewells Hospital, Dundee, UK. Ethical approval was obtained from an independent research ethics committee (18/SS/0160) and the study was registered with the ISRCTN registry (ISRCTN15373978). The study was conducted in accordance with the Declaration of Helsinki, and written informed consent was obtained from all participants before inclusion in the study.

Eligible participants were aged 18–55 years with type 1 diabetes of at least 5 years’ duration and HbA_1c_ <75 mmol/mol (<9%). Participants had confirmed IAH (Gold score ≥4 [2], modified Clarke score ≥4 [12] or Dose Adjustment For Normal Eating [DAFNE] hypoglycaemia awareness rating 2 or 3 [13]) and/or evidence of recurrent hypoglycaemia on flash glucose monitoring. Full eligibility criteria are detailed in the study protocol [11]. Candidates were not preselected based on sex or racial phenotype, and race and ethnicity were determined by investigator observation. The background population in Scotland in adult type 1 diabetes is 55% male and 96% white [14].

Using a web-based system, participants were randomly allocated to real-time continuous glucose monitoring (RT-CGM) or RT-CGM+HIIT. The allocation sequence was generated by an individual not otherwise involved in participant recruitment.

Participants were identified using the Scottish Diabetes Research Network (SDRN) and from diabetes outpatient clinics in NHS Tayside, UK. The study took place at the Clinical Research Centre, Ninewells Hospital and Medical School, Dundee. All people who met the study criteria were offered the chance to participate in the study.

**Fig. 1:**
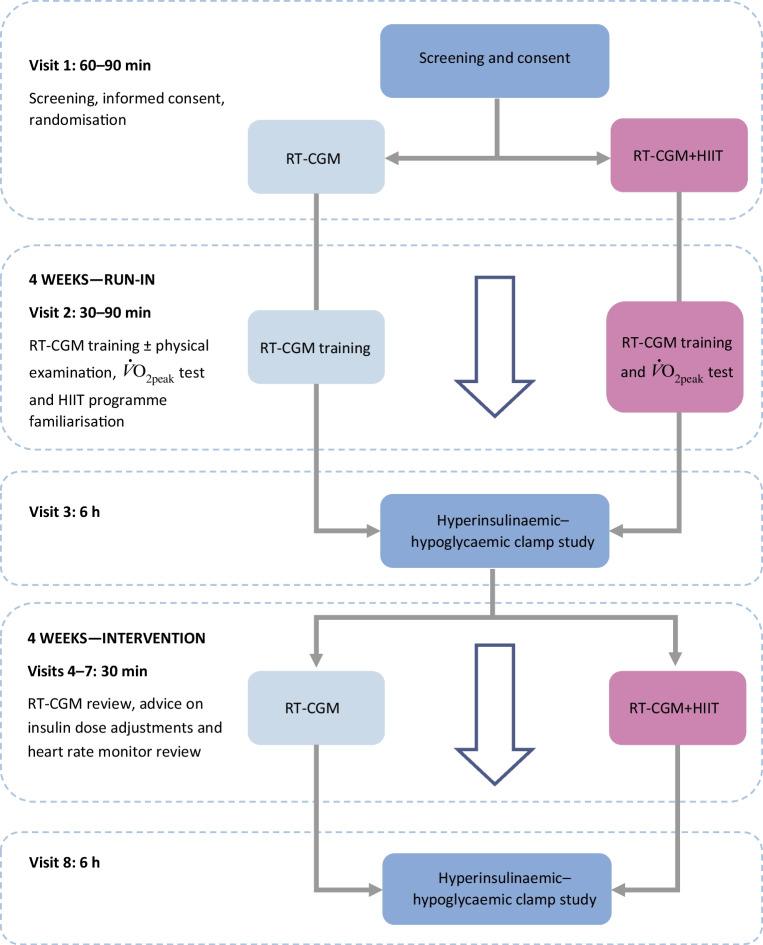
Flow diagram of HIT4HYPOS study visits

After obtaining informed written consent and initial screening, participants were randomised to either 4 weeks of RT-CGM or 4 weeks of RT-CGM+HIIT. Participants attended the Clinical Research Centre for eight study visits in total (Fig. 1). Following randomisation, participants entered a 4 week run-in period to optimise their glycaemic control. They had a weekly telephone call or email from the research fellow to provide advice regarding insulin dose adjustment and hypoglycaemia avoidance. During weeks 1 and 2 of the run-in-period, visit 2 took place. All participants were taught how to use the real-time continuous glucose monitor (Dexcom G6, Dexcom, San Diego, CA, USA). Those in the HIIT group underwent anthropometric measurements, a physical examination and an electrocardiogram. In addition, participants in the HIIT group undertook an incremental maximal to volitional exhaustion exercise test on a cycle ergometer (Corival, Lode, Groningen, the Netherlands; Metamax 3B, CORTEX Biophysik, Leipzig, Germany) to determine their ${\dot{V}\mathrm{O}}_{2\mathrm{peak}}$ and peak heart rate [11].

### Experimental hypoglycaemia

At the end of the run-in period and following the 4 week intervention period, participants underwent a 90 min hyperinsulinaemic–hypoglycaemic (2.5 mmol/l) clamp study (visit 3) as previously described [10, 11]. Arterialised blood for measurement of insulin and counterregulatory hormones (adrenaline [epinephrine], noradrenaline [norepinephrine], glucagon) was taken every 30 min (*t*=−30, 0, 30, 60 and 90 min) during the clamp. At each 30 min time point, participants completed the Edinburgh Hypoglycaemia Symptom Scale (EHS) [15], a validated questionnaire, scoring 11 symptoms from 1 (not at all) to 7 (very severe) on a visual analogue scale. A sequence of psychometric tests known to be sensitive to hypoglycaemia were carried out in the same order, starting approximately 2 min before each 30 min time point: digit symbol substitution test (DSST) [16] and four-choice reaction time (4CRT) [17]. The same clamp procedure was conducted on completion of the 4 week intervention period.

### Intervention

Following the baseline hyperinsulinaemic–hypoglycaemic clamp study, participants entered a 4 week intervention period. All participants used RT-CGM, in addition, those in the HIIT group carried out a 20 min HIIT programme on a stationary exercise bike three times a week. Each exercise session in the HIIT programme consisted of 5 min of gentle warm up cycling followed by 4×30 s cycle sprints (with 2 min active recovery after each sprint) followed by a 5 min cooldown, with the aim of achieving ≥90% of the peak heart rate obtained during the ${\dot{V}\mathrm{O}}_{2\mathrm{peak}}$ test during visit 2. Participants were issued with a heart rate monitor (Polar Vantage M and Polar H10; Polar Electro OY, Coventry, UK) to record their heart rate during the sessions. Standardised advice [18] was given regarding insulin dose adjustment and target blood glucose levels.

During the intervention period, all participants attended a weekly study visit (visits 4–7) to download RT-CGM data and receive advice as required on insulin dosing to optimise blood glucose levels and aid hypoglycaemia avoidance. Heart rate data were downloaded from participants in the HIIT group to ensure that their target heart rate (≥90% peak) was reached during the exercise sessions.

There was a 4 to 7 day break at the end of the active intervention period prior to the second, matched hyperinsulinaemic–hypoglycaemic clamp study (visit 8). This was to avoid any lasting effect from the final exercise session.

### Continuous glucose monitoring

RT-CGM was used in both groups to assess the frequency of exposure to level 1 (3.9–3.0 mmol/l glucose) [19] and level 2 (<3.0 mmol/l glucose) [19] hypoglycaemia during the study period. A hypoglycaemic event was defined according to the glycaemic criteria, duration and descriptive criteria reported in a recent international consensus statement on RT-CGM-derived metrics [19].

### Laboratory assays

Arterialised blood samples were taken every 5 min during the hyperinsulinaemic–hypoglycaemic clamp; these samples were centrifuged and plasma glucose was analysed at the bedside by an enzymatic-amperometric method using chip sensor technology (Biosen C-Line GP+, EKF Diagnostics, Barleben, Germany). Additional samples taken at 30 min intervals were centrifuged within 1 h to separate the serum or plasma and stored at −80˚C before performing the following assays: insulin (Alpco, Salem, NH, USA; CV inter-assay 5.5%, intra-assay 6.4%), adrenaline (Alpco; coefficient of variation [CV] inter-assay 19.6%, intra-assay 14.3%), noradrenaline (Alpco; CV inter-assay 16.3%, intra-assay 12.0%) and glucagon (EMD Millipore, Burlington, MA, USA; CV inter-assay 15.7%, intra-assay 11%). Samples were analysed in duplicate according to the manufacturer’s instructions.

### Data and statistical analysis

The predefined primary outcome was the difference in hypoglycaemia-induced adrenaline responses from baseline following RT-CGM or RT-CGM+HIIT. As this was a pilot study, a formal sample size calculation was not carried out. The aim was to recruit up to 32 participants split equally between the two study groups. Because of the impact of the COVID-19 pandemic, 22 participants were recruited and 18 participants completed the experimental studies. Secondary outcomes examined were the difference in symptom awareness score, cognitive function and other CRR during hypoglycaemia following either RT-CGM or RT-CGM+HIIT. For the primary and secondary endpoints, a generalised estimating equation (GEE) was used, adjusting for baseline and euglycaemia (0 min). A *p* value <0.05 (two-tailed) was considered statistically significant. All data are presented as mean ± SEM. Glucose control was quantified in terms of hypoglycaemic event exposure, mean glucose, SD of glucose and the glucose management indicator (GMI), which were calculated using AGATA [20], a software for automated glucose data analysis. Statistical analyses were conducted using IBM SPSS Statistics 25 (IBM, Armonk, NY, USA). Graphs were created using GraphPad Prism 7.05 (GraphPad Software, San Diego, CA, USA).

## Results

### Participant characteristics

**Fig. 2:**
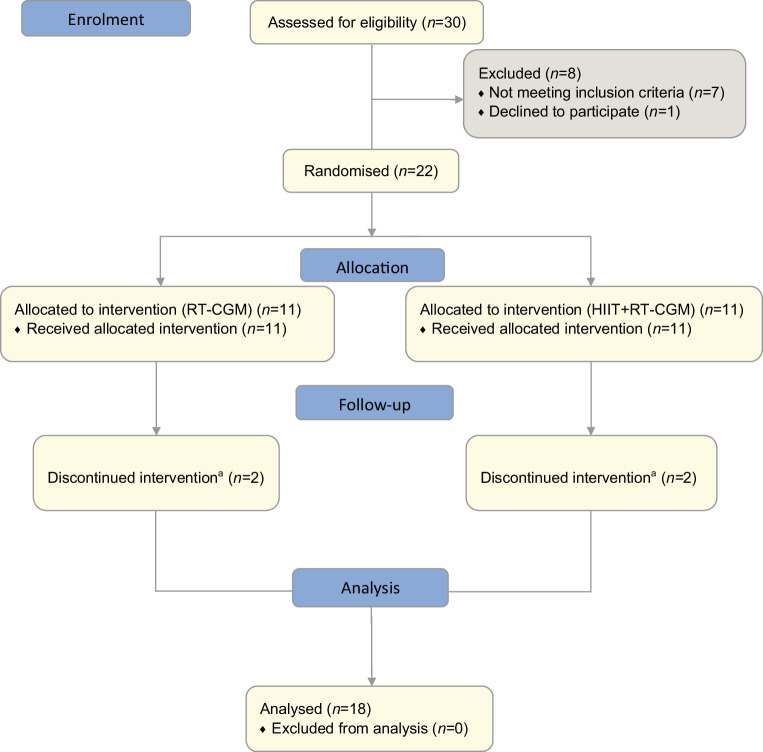
HIT4HYPOS study CONSORT flow diagram. ^a^Reasons for discontinuing intervention: pregnancy, moved away from the area, poor i.v. access or non-compliance

Recruitment was carried out from January 2019 to March 2021. Of the 30 participants screened and consented, seven did not meet the inclusion criteria and one withdrew before commencing the study because of time constraints. Twenty-two participants were randomised but four were withdrawn during the study period as a result of pregnancy (*n*=1), poor i.v. access (*n*=1), moving away from the area (*n*=1) and non-compliance during the run-in period (*n*=1). Therefore, 18 participants completed the study (Fig. 2).

The 18 participants (nine men and nine women, age 20–54 years, all white) who completed the study had long-duration type 1 diabetes [median (IQR) duration 27 (18.75–32) years] and IAH, defined by at least one of a Gold score ≥4 (RT-CGM vs RT-CGM+HIIT: 5/9 vs 5/9), modified Clarke score ≥4 (RT-CGM vs RT-CGM+HIIT: 7/9 vs 8/9) or DAFNE hypoglycaemia awareness rating 2 or 3 (RT-CGM vs RT-CGM+HIIT: 7/9 vs 9/9). Mean (SEM) HbA_1c_ at randomisation was 56 (2) mmol/mol (7.3% [0.2]).

Nine participants randomised to RT-CGM+HIIT were able to complete the intervention and to achieve at least 90% of their peak heart rate obtained during the ${\dot{V}\mathrm{O}}_{2\mathrm{peak}}$ assessment during every session (electronic supplementary material [ESM] Table 1 and ESM Fig. 1).

### Continuous glucose monitoring

Analysis of the RT-CGM data during the 4 week intervention period revealed no significant differences in exposure to all hypoglycaemia (RT-CGM vs RT-CGM +HIIT: mean [SEM] 37 [9] vs 26 [5] episodes, *p*=0.32), level 1 hypoglycaemia (28 [7] vs 22 [4] episodes, *p*=0.45) or level 2 hypoglycaemia (9 [3] vs 4 [1] episodes, *p*=0.29). Similarly, there were no significant differences in mean (SEM) duration of all hypoglycaemia (38 [3] vs 33 [3] min, *p*=0.31); level 1 hypoglycaemia (34 [3] vs 31 [3] min, *p*=0.45) or level 2 hypoglycaemia (22 [2] vs 21 [2] min, *p*=0.68) (ESM Table 2). There were no reported episodes of level 3 hypoglycaemia [21].

Glucose (mean [SEM] 8.7 [0.3] vs 9.2 [0.3] mmol/l, *p*=0.27), SD of glucose (3.1 [0.1] vs 3.4 [0.2] mmol/l, *p*=0.23) and GMI (7.1% [0.1] vs 7.3% [0.1], *p*=0.27) also did not differ significantly between groups (all RT-CGM vs RT-CGM+HIIT, respectively).

### Hyperinsulinaemic–hypoglycaemia clamp studies

Mean (SEM) plasma insulin and glucose levels were consistent between clamp studies, with no significant differences between groups at baseline or after the 4 week intervention (overall *p*=0.36 and *p*=0.50, respectively) (Table 1). Small, but non-significant differences were observed between groups in mean (SEM) glucose infusion rates (60–90 min) required to maintain equivalent hypoglycaemic plateaus (RT-CGM vs RT-CGM+HIIT: 3.33 [0.52] vs 2.76 [0.40] mg^−1^ kg^−1^ min, *p*=0.83; Table1).

**Table 1:** Counterregulatory hormone, glucose, insulin and glucose infusion rates during the hyperinsulinaemic–hypoglycaemic clamp studies

Physiological measure	Pre RT-CGM	Post RT-CGM	Pre RT-CGM+HIIT	Post RT-CGM+HIIT
Adrenaline (pmol/l)				
0 min	295 (43)	242 (54)	899 (450)	302 (59)
90 min	3749 (812)	3398 (346)	4074 (725)	4606 (1055)
Noradrenaline (pmol/l)				
0 min	4092 (901)	4337 (684)	4650 (692)	4327 (554)
90 min	6363 (1050)	5620 (875)	6167 (873)	6358 (962)
Glucagon (ng/l)				
0 min	52 (4)	54 (4)	52 (8)	48 (7)
90 min	58 (5)	54 (6)	58 (6)	70 (8)
Glucose (mmol/l)				
0 min	5.08 (0.06)	4.84 (0.07)	4.91 (0.06)	4.59 (0.06)
90 min	2.53 (0.02)	2.49 (0.03)	2.53 (0.01)	2.50 (0.01)
Insulin (pmol/l)				
0 min	599 (69)	551 (51)	677 (51)	663 (74)
90 min	584 (28)	561 (23)	670 (24)	654 (31)
GIR (mg kg^−1^ min^−1^)				
0 min	4.31 (0.58)	4.92 (0.68)	4.20 (0.49)	4.11 (0.46)
90 min	2.84 (0.46)	3.33 (0.52)	2.81 (0.43)	2.76 (0.40)

Table shows mean (SEM) values under euglycaemic (0 min) and hypoglycaemic (90 min) conditions at baseline (pre) and after the 4 week intervention (post) in the RT-CGM and RT-CGM+HIIT cohorts

GIR, glucose infusion rate

### Hormonal CRR

**Fig. 3:**
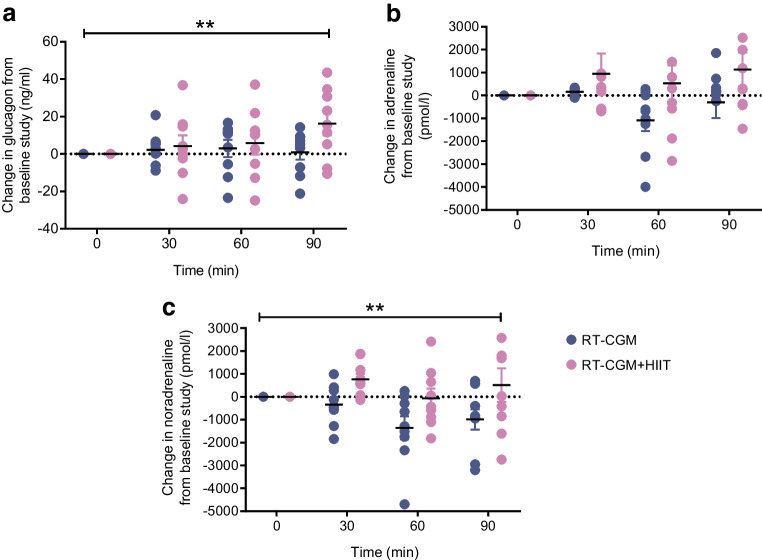
Exposure to 4 weeks of HIIT results in greater counterregulatory hormone responses to subsequent hypoglycaemia. Mean difference adjusted for baseline in plasma glucagon (**a**), adrenaline (**b**) and noradrenaline (**c**) between hyperinsulinaemic–hypoglycaemic clamp studies (*n*=18). Values are mean ± SEM, GEE used. **p<0.01

When comparing the differences in mean (SEM) catecholaminergic responses between the pre- and post-intervention hypoglycaemic clamps (Table 1), the adrenaline response did not differ between groups (RT-CGM vs RT-CGM+HIIT: −298 [687] vs 1130 [747] pmol/l, *p*=0.11), but the noradrenaline response (−988 [447] vs 514 [732] pmol/l, *p*=0.02) was greater following RT-CGM+HIIT (Fig. 3). Relative to the pre-intervention results, there was an increase in plasma glucagon during hypoglycaemia following RT-CGM+HIIT, but not RT-CGM (RT-CGM vs RT-CGM+HIIT: 1 [4] vs 16 [6] ng/l, *p*=0.01; Fig. 3 and Table 1).

### Symptomatic responses

**Fig. 4:**
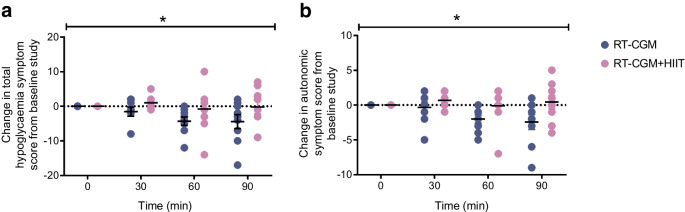
Exposure to 4 weeks of HIIT results in greater counterregulatory symptom responses to subsequent hypoglycaemia. Mean difference adjusted for baseline in total symptom score (**a**) and autonomic symptoms (**b**) between hyperinsulinaemic–hypoglycaemic clamp studies (*n*=18). Values are mean ± SEM, GEE used. *p<0.05

When comparing the difference in mean symptom scores between the pre- and post-intervention hypoglycaemic clamps, significant effects of RT-CGM+HIIT were seen for total and autonomic symptoms (Fig. 4). We found that hypoglycaemia-induced total symptom scores (mean [SEM] 25 [3] vs 20 [2], *p*=0.02) and autonomic symptom scores (13 [2] vs 10 [1], *p*=0.02) were reduced from baseline to post intervention in the control condition, whereas, following 4 weeks of RT-CGM+HIIT, both total (27 [3] vs 25 [2], *p*=0.90) and autonomic (12 [1] vs 12 [1], *p*=0.62) hypoglycaemia-induced symptom scores were maintained between baseline and post intervention (ESM Table 3).

### Cognitive response

No significant differences were found in either the 4CRT or DSST during hypoglycaemia between baseline and post RT-CGM or RT-CGM+HIIT (*p*=0.41 and *p*=0.61, respectively; ESM Table 3).

## Discussion

In this pilot study in people with type 1 diabetes who have IAH we found that, compared with control participants who used RT-CGM alone, participants who underwent 4 weeks of HIIT in combination with RT-CGM had greater noradrenaline, glucagon and total and autonomic symptom responses during a subsequent hypoglycaemia clamp study. In addition, while overall differences between groups were not statistically significant, possibly because of the relatively small number of participants in this pilot trial, there were 29% fewer total hypoglycaemic episodes, 21% fewer level 1 hypoglycaemic episodes and 56% fewer level 2 hypoglycaemic episodes in those undergoing HIIT in combination with RT-CGM compared with those using RT-CGM alone. These findings suggest that there are sustained effects of HIIT on the CRR to hypoglycaemia in people with type 1 diabetes and IAH, at least over a 4 week period, and that HIIT, at the very least, is a safe form of exercise in this cohort who are most at risk of hypoglycaemia. Our study population of adults with type 1 diabetes was typical of those people previously self-reported in Scotland to have IAH in terms of median age and duration of diabetes.

Habituation is a form of adaptive memory described as a reduction of the psychological, behavioural or physiological responses to a stimulus as a result of repeated or prolonged exposure [8, 9]. This definition of a habituated response is consistent with the changes in CRR evoked by recurrent hypoglycaemia in people with type 1 diabetes, in whom there is also a progressive reduction or loss of psychological (anxiety), behavioural (food seeking) and physiological (symptom and hormonal) CRR [8, 9, 22]. Dishabituation occurs when there is an enhancement of the habituated response following the introduction of a novel, usually strong stressor [8]. We have previously shown in an animal model of defective CRR [23] and in humans with type 1 diabetes and IAH [10] that a single exposure to HIIT significantly increases the CRR to hypoglycaemia induced the following day. Similarly, acute cold (4°C) exposure in an animal model of defective CRR [24] was also shown to augment the CRR to subsequent hypoglycaemia [25]. These findings all support the hypothesis that the suppression of CRR that results from exposure to recurrent hypoglycaemia is a form of habituation.

The current study was designed to establish whether people with IAH and type 1 diabetes might adapt to recurrent HIIT in the same way as they do to recurrent hypoglycaemia, which would make this intervention less useful clinically. This is referred to as habituation to the dishabituating stimulus [8]. In our previous study, a single bout of HIIT resulted in greater adrenaline, noradrenaline, glucagon and total and autonomic symptom scores during next-day hypoglycaemia, although only the adrenaline and total symptom responses were statistically significant [10]. In the current study, we also found that the counterregulatory hormonal and symptom responses were greater after 4 weeks of HIIT than in the control arm, although only the noradrenaline, glucagon and total and autonomic symptom responses were statistically significant. It should be noted that only the glucagon response to subsequent hypoglycaemia in the HIIT group was increased relative to the baseline study, with significant differences between groups resulting in part from small decrements from baseline in the control condition. This means that we cannot exclude the possibility that there was some habituation to HIIT per se. However, the increase in adrenaline from baseline following 4 weeks of HIIT, although not significant, was greater in magnitude than in the previous study [10], indicating that the lack of significance may have resulted from the relatively small number of participants studied. Future studies with a greater number of participants will be needed to determine whether there are continuing benefits of HIIT on the adrenaline CRR to hypoglycaemia.

Our results differ from those of previous experimental studies in people with type 1 diabetes that found that moderate intensity exercise led to suppression of the CRR to subsequent hypoglycaemia [26] and similarly that antecedent hypoglycaemia suppressed the CRR to subsequent exercise [25, 26]. However, another feature of habituation is ‘stimulus generalisation’, which occurs when very similar stimuli are presented [8, 9]. This suggests that mild to moderate intensity exercise may show stimulus generalisation with hypoglycaemia, which would also explain the increased risk of nocturnal hypoglycaemia often attributed to exercise [17]. In contrast, when a novel and strong stimulus is presented, as in the case here with HIIT, dishabituation of the habituated response can occur and the risk of subsequent hypoglycaemia is reduced.

The reduced symptom response following 4 weeks of RT-CGM in the control group was unexpected. This change in the symptom response was not accompanied by significant reductions in adrenaline and noradrenaline responses during the second hypoglycaemic clamp, which might have been expected if this was an effect of greater exposure to hypoglycaemia in the control participants. Nevertheless, while differences between groups in total exposure to level 1 and level 2 hypoglycaemia during the 4 week intervention period were not significant, there were fewer hypoglycaemic episodes (21% and 56%, respectively) in the HIIT cohort. Unfortunately, RT-CGM data were not collected prior to randomisation and so it is not clear if RT-CGM use resulted in an increase in participant exposure to hypoglycaemia. Longer-term RT-CGM interventions in people with well-controlled type 1 diabetes and IAH have shown that RT-CGM reduces hypoglycaemia frequency and duration [27], which would be expected to increase rather than decrease CRR responses to hypoglycaemia. However, in that clinical study, despite a 72% reduction in hypoglycaemia incidence after 6 months, there was no improvement in hypoglycaemia awareness scores [27]. This may be because RT-CGM underestimates the occurrence and degree of hypoglycaemia [28] and any ongoing exposure to hypoglycaemia may prevent the restoration of hypoglycaemia awareness. In the present study both groups were started on RT-CGM so it would be anticipated that any impact of this intervention per se would have affected both groups equally. Greater familiarisation of the experimental procedure may have reduced participant anxiety, contributing to the reduced symptom responses, but again both groups would have been affected equally.

An unexpected finding in the present study was the significant increase in plasma glucagon during hypoglycaemia following RT-CGM+HIIT. A small, but non-significant increase in glucagon was also seen during next-day hypoglycaemia after a single bout of HIIT in our previous study [10]. As far as we are aware this is the first time this has been reported in people with type 1 diabetes. The glucagon response to insulin-induced hypoglycaemia is markedly impaired in nearly all people with type 1 diabetes within approximately 5 years of disease diagnosis [29, 30]. The mechanisms that mediate the alpha cell response to hypoglycaemia are thought to involve the paracrine/endocrine influences of other beta and delta cells of the islet, and also sympathetic and parasympathetic innervation as well as circulating adrenaline [31]. In type 1 diabetes it has generally been assumed that the profound defect in alpha cell glucagon secretion is a consequence of autoimmune beta cell destruction, that is, that the loss of a paracrine signal or other beta cell-secreted factor is responsible for this defect [29]. In contrast, the glucagon response to exercise is thought to be mediated, in large part, by increased release of catecholamines, which stimulate alpha cells to secrete glucagon [32, 33]. It is possible that HIIT sensitises pancreatic alpha cells to catecholamines, from either the systemic circulation or the sympathetic nervous system. Experimental research in a rodent model of type 1 diabetes has shown that the glucagon response to insulin-induced hypoglycaemia can be enhanced by activation of key hypothalamic glucose-sensing regions, which in turn amplify the autonomic response to hypoglycaemia [34]. However, to date, most interventions that have improved the autonomic response to hypoglycaemia in people with type 1 diabetes and IAH, such as hypoglycaemia avoidance [5], have not shown a similarly enhanced glucagon response. This could imply that there may be a signal released by exercising muscle that acts either directly on the pancreas, increasing beta-adrenergic sensitivity, and/or potentially within brain stem or hypothalamic integrative centres, resulting in the partial restoration of a glucagon response to insulin-induced hypoglycaemia in type 1 diabetes. Additional studies will be required to examine this further.

There are a number of limitations to this study. The hyperinsulinaemic–hypoglycaemic clamp is the gold standard technique for assessing CRR to hypoglycaemia [35]. However, as this is conducted under strictly controlled conditions in a laboratory, it is unlikely to fully represent real-world experiences. The small sample size in this study is also a limitation. Unfortunately, the COVID-19 pandemic had a detrimental effect on the study. The study was suspended twice, which led to considerable delays, and many individuals were hesitant to attend the hospital clinical research centre, meaning that recruitment and retention were very challenging. There were also delays due to self-isolation. The HIIT programme was carried out on a stationary exercise bike, which was difficult for participants to maintain with the closure of gyms. The study protocol was therefore amended to enable the provision of exercise bikes to participants’ homes. In addition, local and national guidelines limited travel, which had an impact on those coming from further afield for visits. To ensure participant safety, additional COVID-19 screening calls were required before each visit and the need for personal protective equipment use by the team and participants at every visit increased participants’ anxiety.

In conclusion, in this pilot clinical trial we have shown that a 4 week programme of HIIT using a cycle ergometer in combination with RT-CGM for people with type 1 diabetes who have IAH improves aspects of the CRR to hypoglycaemia in comparison to the use of RT-CGM alone. Importantly, we found that the glucagon CRR to hypoglycaemia was improved by HIIT both relative to the control condition and baseline. The unexpected HIIT-mediated amplification of the glucagon response to insulin-induced hypoglycaemia is a potentially important finding that warrants further investigation, given that the glucagon response to insulin-mediated hypoglycaemia is almost universally absent in people with type 1 diabetes of >5 years’ duration. Although our findings need to be explored further and replicated in studies of longer duration, they suggest that HIIT-mediated dishabituation, at least over a 4 week period, may remain beneficial to people with type 1 diabetes and IAH. In addition, the study also suggests that HIIT programmes may be safely implemented in type 1 diabetes, even in those individuals most at risk of hypoglycaemia.

### Supplementary Information

Below is the link to the electronic supplementary material.

ESM (PDF 363 KB)

## Data Availability

Data are available on request from RJM, who is the guarantor of this work.

## Abbreviations

4CRT: Four-choice reaction time
CRR: Counterregulatory response
DAFNE: Dose Adjustment For Normal Eating
DSST: Digit symbol substitution test
GEE: Generalised estimating equation
GMI: Glucose management indicator
HIIT: High intensity interval training
IAH: Impaired awareness of hypoglycaemia
RT-CGM: Real-time continuous glucose monitoring
