# Supplementary material for: High intensity interval training as a novel treatment for impaired awareness of hypoglycaemia in people with type 1 diabetes (HIT4HYPOS): a randomised parallel-group study
Source: Diabetologia. 2023 Nov 27;67(2):392–402. doi: 10.1007/s00125-023-06051-x (PMC10789679; doi:10.1007/s00125-023-06051-x)

ESM Table 1: Participant heart rate (HR) data. Baseline pre VO_2 Peak test, Target HR (90% Maximum obtained during VO_2 Peak test), Maximum HR reached during each HIT session. All data in bpm.

	RESTING HR	MAX HR DURING VO_2 PEAK TEST	90% MAX HR	PEAK HR PER HIT SESSION											
				**1**	**2**	**3**	**4**	**5**	**6**	**7**	**8**	**9**	**10**	**11**	**12**
**01**	80	182	164	183	183	182	179	185	181	186	179	179	184	176	177
**03**	66	183	165	175	171	173	172	171	173	171	170	170	169	171	170
**06**	89	200	180	193	192	188	185	186	188	184	185	188	187	186	184
**08**	89	175	158	171	171	168	172	166	168	165	167	164	166	169	165
**09**	73	170	153	169	162	165	158	162	157	178	159	157	158	157	156
**12**	44	171	154	176	173	171	174	166	222	164	164	164	163	168	167
**16**	86	163	147	161	163	156	158	156	158	158	161	154	160	157	156
**20**	87	171	154	163	160	159	157	157	156	157	157	157	160	157	159
**22**	71	173	156	178	171	172	172	174	169	177	170	170	169	168	175

ESM Table 2. Continuous glucose monitoring measures during 4-week HIIT+CGM and control CGM-alone study conditions.

Metric (unit)	CGM	HIIT+CGM	p-value
T_{Hypo} *(%)*	3.12 [0.70]	2.17 [0.49]	0.280
$T_{Hypo\ (L1)}$ *(%)*	2.65 [0.58]	1.92 [0.45]	0.333
$T_{Hypo\ (L2)}$ *(%)*	0.48 [0.15]	0.25 [0.07]	0.191
T_{Hyper} *(%)*	32.63 [3.95]	36.63 [3.28]	0.447
$T_{Hyper(L1)}$ *(%)*	25.42 [2.78]	26.06 [1.98]	0.855
$T_{Hyper\ (L2)}$ *(%)*	7.21 [1.42]	10.57 [2.14]	0.209
T_{Target} *(%)*	64.24 [3.60]	61.20 [3.20]	0.536
$T_{TightTarget}$ *(%)*	39.62 [4.03]	36.78 [2.82]	0.571

ESM Table 3. Symptom responses and cognitive function assessments during all hyperinsulinaemic hypoglycaemic clamps studies. Table shows mean (SEM) values under euglycaemic (0 min) and hypoglycaemic (90min) conditions at baseline and after the 4-week intervention in RT-CGM and RT-CGM+HIIT cohorts.

	**Baseline RT-CGM**	**Post RT-CGM**	**Baseline RT-CGM+HIIT**	**Post RT-CGM+HIIT**
	Mean (SEM) Responses During Hypoglycaemia			
**Symptoms- Edinburgh Hypoglycaemia Score**				
**Total**				
0 min	16 [1]	16 [2]	16 [3]	15 [1]
90 min	25 [3]	20 [2]	27 [3]	25 [2]
**Autonomic**				
0min	7 [2]	6 [1]	6 [1]	6 [0]
90 min	13 [2]	10 [1]	12 [1]	12 [1]
**Cognition**				
**Digit Symbol Substitution Test**				
0 min	52 [2]	54 [3]	45 [3]	47 [4]
90 min	43 [3]	46 [3]	38 [3]	39 [3]
**4-Choice Reaction Time Test (ms)**				
0 min	534 [21]	564 [19]	497 [14]	537 [22]
90 min	623 [20]	634 [211]	591 [32]	612 [24]

ESM Figure. 1: *Heart rate response to HIT. Heart rate (HR) at rest, circles; Target HR, squares; Maximum HR reached, triangles. Maximum HR is mean of all 12 sessions. n = 9, values are mean ± SEM.*

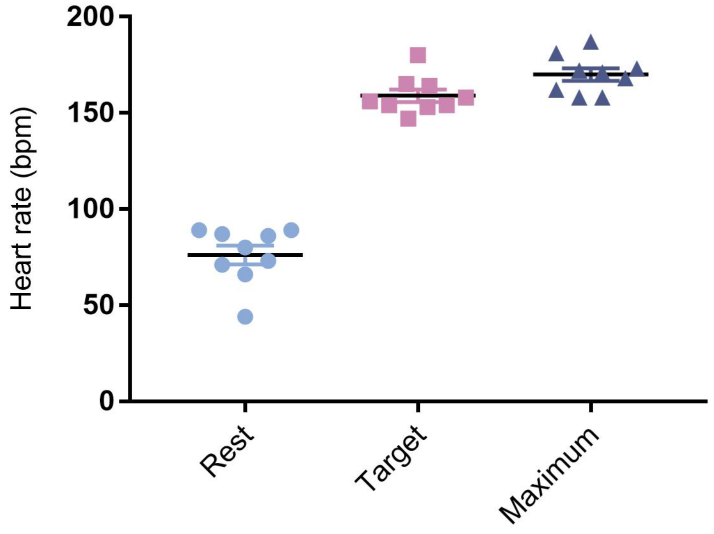

Supplement: Supplementary file 1 — ESM (PDF 363 KB) [file 125_2023_6051_MOESM1_ESM.pdf]
